# The role of calcium stores in long-term potentiation and synaptic tagging and capture in mouse hippocampus

**DOI:** 10.1098/rstb.2023.0241

**Published:** 2024-07-29

**Authors:** Laura A. Koek, Thomas M. Sanderson, John Georgiou, Graham L. Collingridge

**Affiliations:** ^1^ Lunenfeld-Tanenbaum Research Institute, Mount Sinai Hospital, Sinai Health System, Toronto, Ontario M5G 1X5, Canada; ^2^ Department of Physiology, University of Toronto, Toronto, Ontario M5S 1A8, Canada; ^3^ Tanz Centre for Research in Neurodegenerative Diseases, University of Toronto, Toronto, Ontario M5S 1A8, Canada

**Keywords:** synapse, plasticity, metaplasticity, heterosynaptic, associative, memory

## Abstract

The roles of Ca^2+^-induced calcium release in synaptic plasticity and metaplasticity are poorly understood. The present study has addressed the role of intracellular Ca^2+^ stores in long-term potentiation (LTP) and a form of heterosynaptic metaplasticity known as synaptic tagging and capture (STC) at CA1 synapses in mouse hippocampal slices. The effects of two compounds, ryanodine and cyclopiazonic acid (CPA), were examined on LTP induced by three distinct induction protocols: weak (w), compressed (c) and spaced (s) theta-burst stimulation (TBS). These compounds did not significantly affect LTP induced by the wTBS (one episode of TBS; 25 stimuli) or cTBS (three such episodes with a 10 s inter-episode interval (IEI); 75 stimuli) but substantially inhibited LTP induced by a sTBS (10 min IEI; 75 stimuli). Ryanodine and CPA also prevented a small heterosynaptic potentiation that was observed with the sTBS protocol. Interestingly, these compounds also prevented STC when present during either the sTBS or the subsequent wTBS, applied to an independent input. All of these effects of ryanodine and CPA were similar to that of a calcium-permeable AMPA receptor blocker. In conclusion, Ca^2+^ stores provide one way in which signals are propagated between synaptic inputs and, by virtue of their role in STC, may be involved in associative long-term memories.

This article is part of a discussion meeting issue ‘Long-term potentiation: 50 years on’.

## 1. Introduction

The importance of Ca^2+^ signalling for many forms of synaptic plasticity, such as long-term potentiation (LTP) in the hippocampus, is well established [1–3]. In the case of NMDA receptor (NMDAR)-mediated synaptic plasticity, the initial Ca^2+^ signal is due to direct permeation through NMDARs [1,4,5]. The synaptic Ca^2+^ signal can then be magnified through Ca^2+^-induced calcium release (CICR) [5–8]. However, the role of this amplification of the Ca^2+^ trigger for LTP is poorly understood. For example, interfering with Ca^2+^ stores inhibits the induction of LTP [9–11], though not invariably [12,13], and may even promote LTP under certain conditions [11,14,15]. One possible explanation for this inconsistency is that the distinct forms of synaptic potentiation that coexist may have different dependencies on CICR. In this context, at Schaffer collateral commissural (SCC) to CA1 synapses in the hippocampus, where most studies of synaptic plasticity have been performed, NMDAR-dependent synaptic potentiation comprises multiple, mechanistically distinct forms, which includes short-term potentiation (STP) and at least two forms of LTP (LTP1 and LTP2) [16]. STP is induced rapidly and decays in an activity-dependent manner. LTP1 requires the activation of kinases, such as CaMKII. LTP2 requires activation of calcium-permeable AMPA receptors (CP-AMPARs), PKA and de novo protein synthesis [16,17]. Interestingly, LTP2, unlike STP and LTP1, can be associated with a small heterosynaptic potentiation [18]. In the present study, we have investigated the role of CICR in these various forms of synaptic potentiation, when triggered by theta-burst stimulation (TBS).

In addition to STP and LTP at SCC-CA1 synapses, a process known as synaptic tagging and capture (STC) exists [19], which is a form of heterosynaptic metaplasticity that also requires activation of PKA, protein synthesis [18,20–23] and CP-AMPARs [18,24]. STC describes how induction of LTP at one set of synapses, using a strong induction protocol (‘strong stimulus’), can influence the threshold, magnitude and/or longevity of homosynaptic LTP induced at a separate set of synapses by a weaker induction protocol (‘weak stimulus’) [19,25]. Understanding the underlying mechanisms of this form of metaplasticity is important since STC may explain the process by which long-term memory is formed and consolidated [26,27]. While many molecules participate in the STC process [28], we have recently proposed that the tag is primarily a set of CP-AMPARs and presented a modified hypothesis [18,24]: during a ‘strong stimulus’ (in our case a spaced TBS; sTBS), the first episode drives CP-AMPARs into the plasma membrane at perisynaptic sites, and subsequent episodes drive these CP-AMPARs into the synapse, where they are required to trigger LTP2. Critically for this hypothesis, CP-AMPARs are also driven into the perisynaptic plasma membrane of neighbouring naive synapses (i.e. ones that did not receive TBS). From here, they may be immediately trafficked into the synapse to generate heterosynaptic potentiation. Alternatively, they can dwell at perisynaptic loci for a period of time. In response to a ‘weak stimulus’ (in our case, the weak TBS; wTBS) that would by definition only generate LTP1, these perisynaptic CP-AMPARs are driven into the synapse to generate LTP2. In this scheme, the ‘synaptic tag’ comprises the perisynaptic CP-AMPARs and the ‘capture’ relates to the process that drives these CP-AMPARs into the synapse in response to a ‘weak stimulus’.

Interestingly, previous work has demonstrated the potential for calcium stores to prime LTP [29] and to enhance both the level of STC [30] and the time window for the interaction between the two inputs. In the present study, the role of CICR in both LTP and STC has been further investigated. The findings reveal that CICR is not required for STP or LTP1 but is involved in LTP2, heterosynaptic potentiation and STC. Therefore, the role of CICR corresponds with the roles of CP-AMPARs, PKA and de novo protein synthesis, processes required for long-lasting, persistent forms of memory. As STC mechanisms enable temporal and spatial integration of information within dendrites, a model for the role of CICR in these associative forms of memory through synaptic plasticity and metaplasticity is proposed.

## 2. Methods

### (a) Subjects

Experiments were performed as described in [17]. Briefly, C57BL/6J male mice (10–12 weeks of age) were anaesthetized with isoflurane and euthanized by decapitation in accordance with the Canadian Council on Animal Care (CCAC) guidelines and an AUP approved by The Centre for Phenogenomics (Toronto, Ontario, Canada) Animal Care Committee. Transverse hippocampal slices (400 µm) were prepared using a vibratome (VT1200S; Leica Biosystems, Ontario, Canada). The cutting solution for the dissection contained (mM): 124 NaCl, 3 KCl, 26 NaHC0_3_, 1.25 NaH_2_PO_4_, 10 D-Glucose and 5 MgSO_4_ and 1 CaCl_2_. The CA3 region was cut with a scalpel blade to reduce upstream neuronal excitability and slices were transferred to an incubation chamber containing artificial cerebrospinal fluid (ACSF) recording solution containing (mM): 124 NaCl, 3 KCl, 26 NaHCO_3_, 1.25 NaH_2_PO_4_, 2 MgSO_4_, 10 D-Glucose and 2 CaCl_2_ (bubbled with 95% O_2_ and 5% CO_2_). Slices were allowed to recover at 32°C for 30 min after sectioning and for a minimum of 1 h at 22°C before recordings were obtained.

### (b) Electrophysiology

Hippocampal slices were perfused at 2 ml min^−1^ with the oxygenated ACSF at 30°C. Two bipolar electrodes were positioned in the stratum radiatum on either side and equidistant to a recording electrode. Two independent SCC pathways were stimulated alternately using a constant current stimulator (0.033 Hz frequency, pulse width 0.1 ms; STG 4002; Multi Channel Systems, Kusterdingen, Germany) to evoke synaptic responses. Signals were amplified with a Multiclamp 700B (Molecular Devices, San Jose, CA, USA) and digitized with a BNC-2110 (National Instruments, Austin, TX, USA) A/D board, at a sampling rate of 20 kHz. The independence of the two inputs was verified by the absence of heterosynaptic paired-pulsed facilitation, using an inter-pulse interval of 50 ms. Throughout the experiments, paired pulses were applied within each pathway (50 ms inter-stimulus interval). The initial slope of the evoked field excitatory postsynaptic potential (fEPSP) (V s^−1^) was monitored and analysed using WinLTP 11 (WinLTP Ltd, UK) [31].

Following a stable baseline of at least 20 min, LTP was induced using TBS delivered at the same basal stimulus intensity and pulse width. An episode of TBS comprised five bursts at 5 Hz with each burst composed of five pulses at 100 Hz (i.e. 25 pulses in total). A wTBS comprised one episode of TBS (25 pulses). A compressed TBS (cTBS) comprised three TBS episodes, delivered with an inter-episode interval (IEI) of 10 s (75 pulses in total). A sTBS comprised three TBS episodes, with an IEI of 10 min (75 pulses in total). Representative sample traces are an average of five consecutive responses, collected from typical experiments. Each experiment was conducted from hippocampal slices of different animals, hence the *n*-value represents both the number of slices and animals used.

The protocol for studying heterosynaptic metaplasticity was similar to that used to first describe STC [19]. A strong stimulus (in our case a sTBS) was delivered to one input (denoted *S*0) and 30 min later a weak stimulus (in our case a wTBS) was delivered to a second, independent input (*S*1).

### (c) Statistical analyses

All treatment groups were interleaved with control experiments. Data are presented as mean ± s.e.m. In all time-course plots shown, fEPSPs were normalized to the average of the initial 10 min of fEPSP data. Bar plots and cumulative distribution plots quantify the synaptic fEPSP data averaged over the final 10 min of recording. In any other instances, the time (*t*) in minutes refers to a 10 min bin of data centred over the indicated time. Statistical significance was determined using a two-tailed Student’s *t*‐test or one-way ANOVA followed by *post hoc* Dunnett’s multiple comparisons test or Tukey’s test as appropriate. All statistical tests were performed using GraphPad Prism (v. 10.0.03 for macOS, GraphPad Software, Boston, MA, USA). Level of significance within the figures is denoted as follows: *p < 0.05; **p < 0.01; ***p < 0.001.

### (d) Compounds

The compounds used were cyclopiazonic acid (CPA; Hello Bio Inc, Princeton, NJ, USA), IEM-1460 (IEM; Hello Bio Inc) and ryanodine (Hello Bio Inc). Drugs were prepared from frozen stock solutions dissolved in water (CPA, IEM) or dimethylsulfoxide (0.04% DMSO; ryanodine), aliquoted and stored at −30°C, thawed prior to use and diluted into ACSF at least 20 min before their bath application.

## 3. Results

### (a) Ryanodine selectively inhibits LTP2

**Figure 1.**
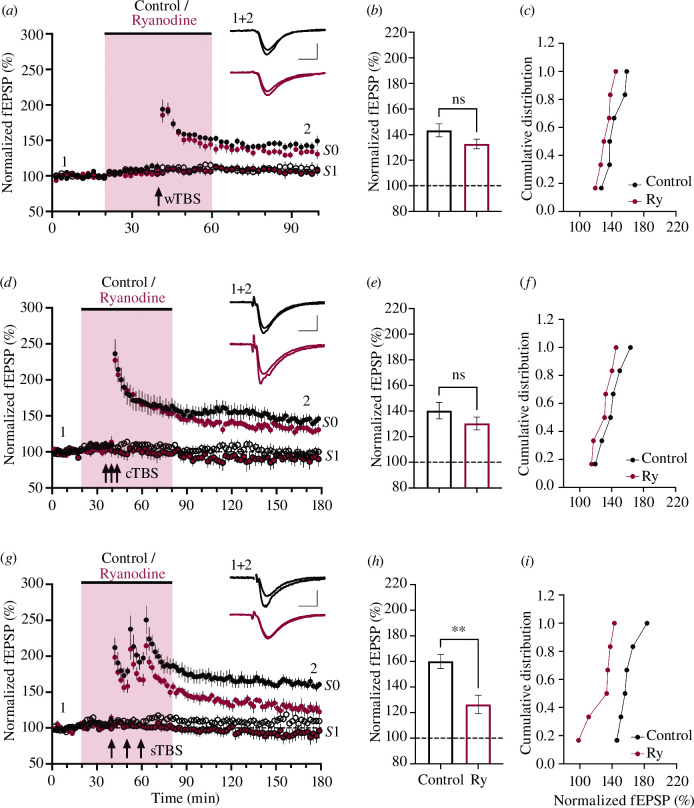
Ryanodine inhibits LTP induced by sTBS. (*a*) Time-course plot showing that 10 µM ryanodine (red symbols) did not significantly affect LTP induced by a wTBS at input *S*0 (black filled circles). The control independent input (*S*1, open circles) was also unaffected. (*b*) The mean (± s.e.m.) level of LTP at input *S*0. (*c*) Cumulative distribution plot of the LTP at input *S*0, plotting each individual experiment. (*d–f*) Equivalent experiments for cTBS. (*g–i*) Equivalent experiments for sTBS. In these and subsequent figures, data from controls are black or white open circles (*S*0 and *S*1 inputs, respectively) and data from treated slices are colour coded. The duration of compound application is denoted by the shaded rectangle. Traces are the average of five consecutive responses obtained from a representative experiment at the times indicated by labels ‘1’ and ‘2’. Calibration bars: 1 mV/5 ms. *p < 0.05, **p < 0.01, ***p < 0.001 (see text for precise *p*-values).

The effect of ryanodine (10 µM) was examined on LTP induced by three distinct induction protocols. In all cases, LTP was preceded by STP, a short-lived form of synaptic potentiation that decays in the order of minutes in an activity-dependent manner [16,32]. STP appeared unaffected by treatment with ryanodine (figure 1). Ryanodine also had no significant effect on LTP1 induced by a wTBS (figure 1*a–c*). For example, quantified at 60 min post-induction, the level of LTP (expressed as % baseline) was 143 ± 5% (*n* = 6) and 133 ± 4% (*n* = 6) in vehicle- and ryanodine-treated slices, respectively (*p* = 0.119). Similarly, ryanodine had no significant effect on LTP1 induced by a cTBS (figure 1*d–f*). For example, at 120 min post-induction, the levels of LTP were 140 ± 6% (*n* = 6) and 130 ± 5% (*n* = 6), respectively (*p* = 0.248). In contrast, ryanodine substantially reduced the level of LTP induced by the sTBS protocol (figure 1*g–i*); a protocol that induces LTP1 plus LTP2 [17]. At 120 min post-induction, the levels of LTP were 160 ± 5% (*n* = 6) and 126 ± 7% (*n* = 6), respectively (*p* = 0.004). Thus, ryanodine specifically reduced LTP induced by sTBS, most probably by selective inhibition of LTP2.

With respect to the wTBS and cTBS experiments, in the control (*S*1 input) pathway, which did not receive any TBS, the synaptic responses were stable throughout (figure *1a,d*). In contrast, in response to sTBS, there was sometimes a small heterosynaptic potentiation, as noted previously [17]. This heterosynaptic potentiation was absent in the ryanodine experiments (figure 1*g*). For example, 30 min post-induction, the levels of heterosynaptic potentiation were 111 ± 4% (*n* = 6) and 99 ± 5% (*n* = 6), in control and ryanodine experiments, respectively.

### (b) CPA selectively inhibits LTP2

**Figure 2.**
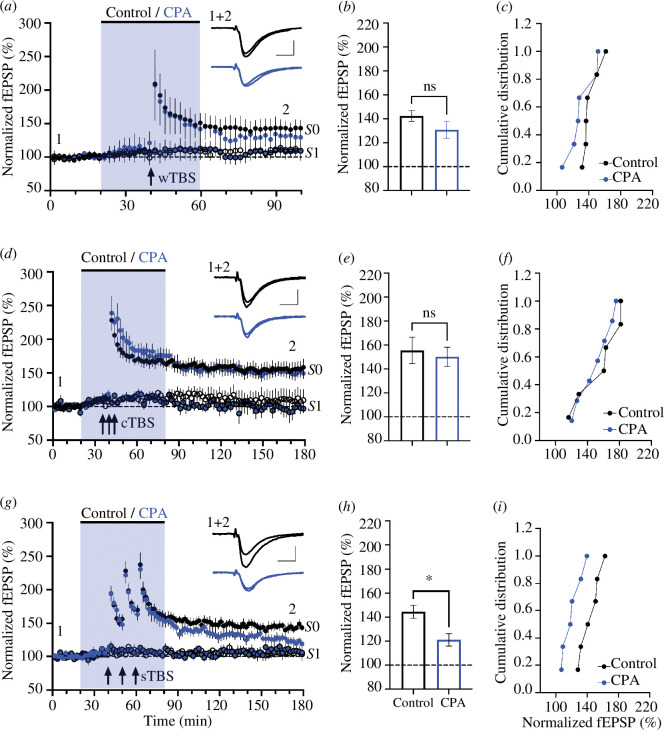
CPA inhibits LTP induced by sTBS. (*a*) Time-course plot showing that 30 µM CPA (blue symbols) did not significantly affect LTP induced by a wTBS (at input *S*0). The control input (*S*1) was also unaffected. (*b*) The mean (± s.e.m.) level of LTP. (*c*) Cumulative distribution plot. (*d–f*) Equivalent experiments for cTBS. (*g–i*) Equivalent experiments for sTBS.

CPA had similar effects to ryanodine (figure 2). In all cases, STP appeared unaffected by treatment with CPA (30 µM). Similarly, CPA did not significantly affect LTP induced by the wTBS; the levels of LTP were 142 ± 5% (*n* = 6) and 133 ± 6% (*n* = 6) in vehicle- and CPA-treated slices, respectively (figure 2*a–c*; *p* = 0.198). Furthermore, CPA did not alter the magnitude of LTP1 induced by the cTBS; the levels of LTP were 156 ± 11% (*n* = 6) and 150 ± 8% (*n* = 7), respectively (figure 2*d–f* ; *p* = 0.698). In contrast, CPA substantially reduced the level of LTP induced by the sTBS protocol; the levels of LTP were 145 ± 5% (*n* = 6) and 121 ± 5% (*n* = 6; figure *2g*–*i*; *p* = 0.011).

### (c) The effects of a CP-AMPAR inhibitor and ryanodine converge on LTP2

**Figure 3.**
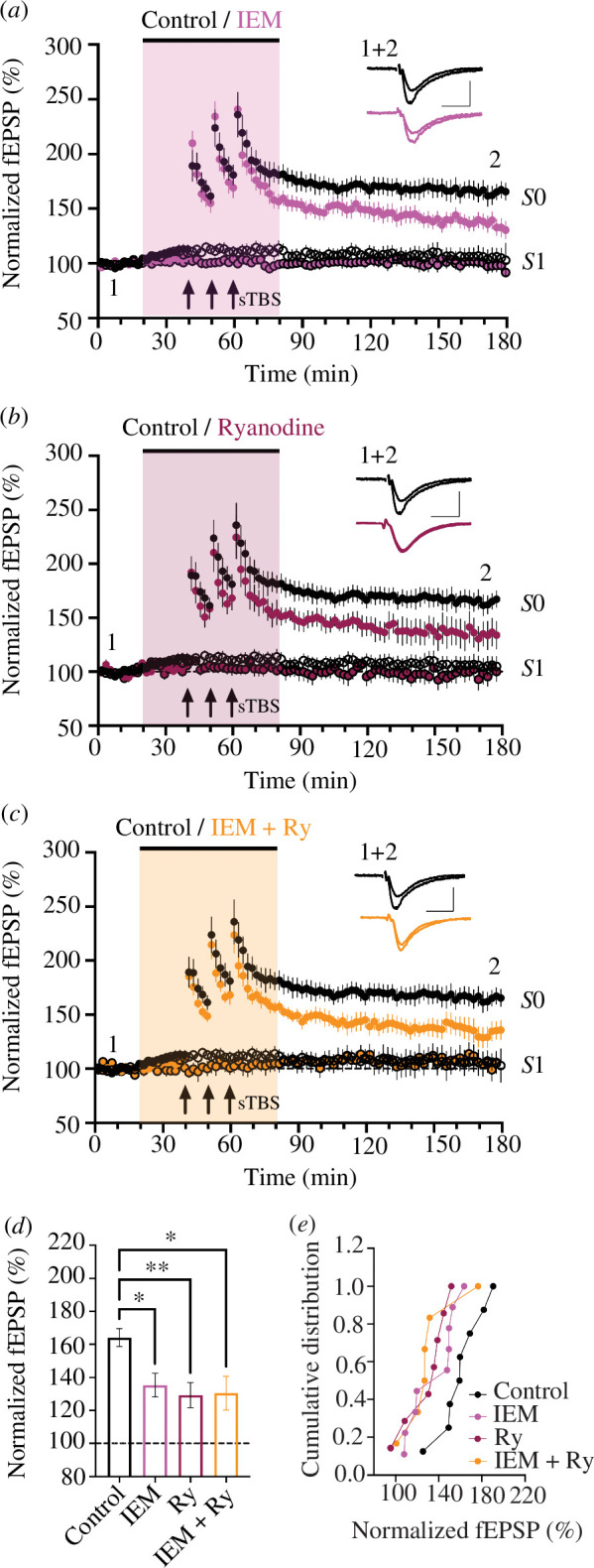
The effects of ryanodine and IEM-1460 are similar and non-additive. Time-course plot showing that (*a*) 30 µM IEM-1460 (pink), (*b*) 10 µM ryanodine (red) and (*c*) the combined application of IEM-1460 and ryanodine (orange) inhibited LTP (induced by a sTBS) to a similar extent. (*d*) The mean (± s.e.m.) level of LTP. (*e*) Cumulative distributions.

Previous work using multiple inhibitors and electrophysiological measures demonstrated that the transient insertion and activation of CP-AMPARs following sTBS is required for LTP2 but not LTP1 [17,33]. The finding that calcium stores are also needed for LTP2 suggests a convergence of action. To test the interaction hypothesis directly, the effects of IEM-1460 (30 µM), an inhibitor of CP-AMPARs [17] and ryanodine (10 µM), applied either alone or in combination were compared in interleaved experiments (figure 3).

As reported previously [17], IEM-1460 inhibited LTP2 (figure 3*a*). Furthermore, consistent with the data presented in figure 1*g–i*, ryanodine inhibited LTP2 in another cohort of hippocampal slices (figure 3*b*). The effect of the combined treatment (figure 3*c*) was similar to the effect of either compound alone (figure 3*a,b*). For example, compared with the level of control LTP (164 ± 5%, *n* = 8), IEM-1460, ryanodine and the combination of ryanodine plus IEM-1460, measured 120 min following sTBS, were 135 ± 7% (*n* = 9, *p* = 0.021), 129 ± 8% (*n* = 7, *p* = 0.008) and 131 ± 10% (*n* = 6, *p* = 0.015), respectively (figure 3*d*). The combined application of ryanodine and IEM-1460 did not result in greater inhibition of LTP2 compared with when either compound was applied singly (*p* = 0.844, one-way ANOVA). These findings further show that the activation of CP-AMPARs and CICR are both required for the generation of LTP2. As the effect of inhibiting each process is not additive, it is concluded that they are involved in the same LTP2 signalling system.

### (d) Role of CP-AMPARs in STC

**Figure 4.**
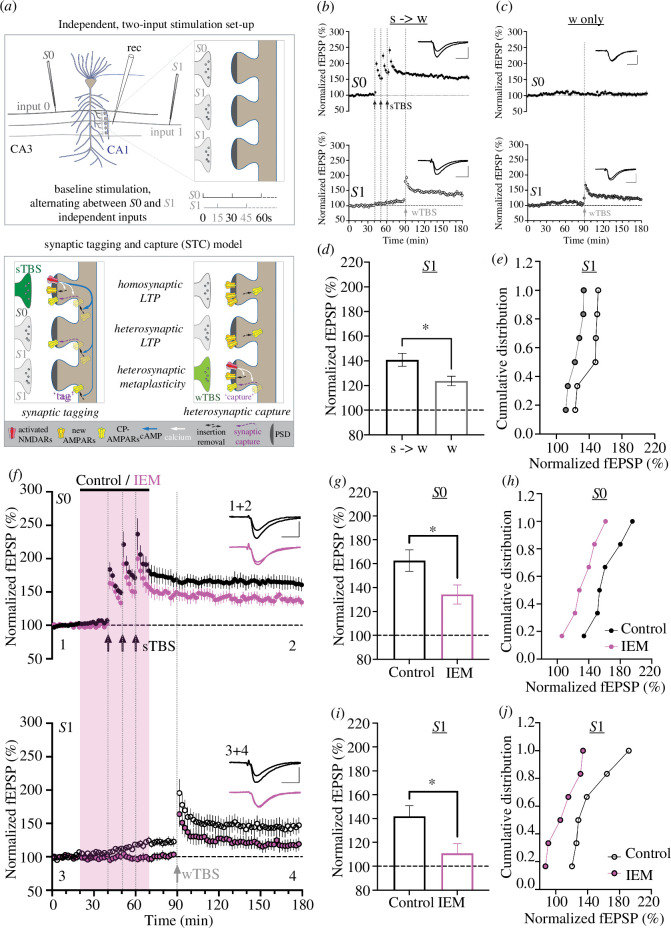
Role of CP-AMPARs in heterosynaptic potentiation and STC. (*a*) Schematic of the two-input experimental arrangement (upper) and mechanisms (lower). As proposed previously [18,24], CP-AMPARs have three distinct roles: firstly, they are required for the generation of homosynaptic, protein synthesis-dependent LTP (i.e. LTP2); secondly, they are inserted into the *synaptic* plasma membrane of some inputs that did not receive sTBS, where they generate protein synthesis-dependent heterosynaptic potentiation (i.e. LTP2); and thirdly, they are inserted into the *perisynaptic* plasma membrane of some additional independent inputs that did not receive the sTBS where they ‘tag’ synapses; a weak stimulus to these inputs (here a wTBS) drives these perisynaptic CP-AMPARs into the synapse, where they trigger protein synthesis-dependent LTP (i.e. LTP2). (*b*) STC control experiments where sTBS at input *S*0 (upper trace) precedes the wTBS at input *S*1 (lower trace) by 30 min (i.e. primed). (*c*) In unprimed LTP, the wTBS (input *S*1) is not preceded by a sTBS (input *S*0). Note LTP at input *S*1 is greater in the primed input (*b*) compared with the unprimed input (*c*); priming-induced enhancement comprises two components: (i) heterosynaptic potentiation and (ii) STC. (*d*) Quantification of input *S*1 LTP for six separate experiments with (*e*) cumulative distribution plots. (*f–j*) Pooled data for equivalent experiments but where IEM-1460 (30 µM) was applied during the sTBS induction protocol (pink). Note that IEM reduced LTP (at input S0) and eliminated the effects of priming of LTP (at input *S*1). Pooled averages and cumulative distribution for input *S*0 (*g,h*) and *S*1 (*i,j*).

In the classical view of STC, the ‘strong’ stimulus (here delivered to input *S*0) generates plasticity-related proteins/products (PRPs) that are captured by tagged synapses (here innervated by input *S*1). To study STC, a sTBS was delivered to one pathway (input *S*0), and 30 min later a wTBS was delivered to a second pathway (input *S*1), defined as independent based on the absence of heterosynaptic paired-pulse facilitation [18]. The set-up is shown in figure 4*a* (upper) together with a simplified model that may explain the role of CP-AMPARs in STC (figure 4*a*, lower). In control slices, LTP was 157 ± 5% and 140 ± 5% (n = 6) at input *S*0 and *S*1, respectively (figure 4*b*). In contrast, the level of LTP induced at input *S*1 without prior sTBS application to *S*0 was 124 ± 4% (figure 4*c*). This difference in the level of LTP at input *S*1 is quantified in figure 4*d,e* (*n* = 6, *p* < 0.025). The enhanced level of LTP observed at input *S*1, when preceded by a sTBS to input *S*0, can be attributed to a mixture of heterosynaptic metaplasticity (STC) and heterosynaptic potentiation (i.e. increase at input *S*1 synaptic responses before the delivery of the wTBS). These results confirm in mice our previous observations made in rat CA3-CA1 synapses [18,24].

As reported previously [17,18,33], IEM-1460 inhibited LTP2 at input *S*0 (figure 4*f*), consistent with the observations reported in figure 3*a,d*. The levels of LTP, quantified 2 h after the third TBS episode, were 163 ± 9% (*n* = 6) and 134 ± 8% (*n* = 6), respectively (*p* = 0.041; figure 1*f*–*h*). IEM-1460 also reduced LTP at input *S*1; at 90 min following the wTBS, the level of LTP was 142 ± 9% (*n* = 6) and 111 ± 8% (*n* = 6), respectively (*p* = 0.027; figure 4*f,i,j*). This reduced level of LTP at the *S*1 input was indistinguishable from the LTP observed when a wTBS was delivered, under control conditions, to input *S*1 without prior application of sTBS to input *S*0 (*p* = 0.656). Note that IEM also eliminated the heterosynaptic potentiation, which is evident at input *S*1 during and following the delivery of sTBS to input *S*0 (figure 4*f*). When *S*1 was quantified at 85 min, the level of heterosynaptic potentiation in control versus IEM-treated slices was 122 ± 6% versus 102 ± 5%, respectively (*p* = 0.030). These data are consistent with the hypothesis that activation of CP-AMPARs during the ‘strong stimulus’ (here induced via sTBS) is required for both heterosynaptic potentiation and STC [18,24].

### (e) Ryanodine prevents STC

**Figure 5.**
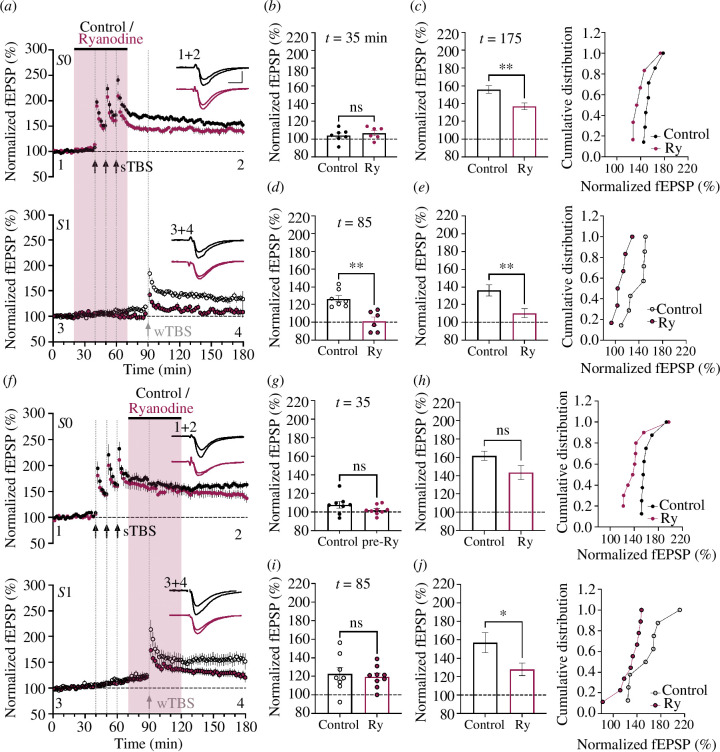
Role of ryanodine receptors in heterosynaptic potentiation and STC. (*a*) Time-course plot showing that 10 µM ryanodine, applied during the sTBS (light red period), inhibits homosynaptic LTP (at input *S*0) and heterosynaptic potentiation and STC (at input *S*1). Analysis for baseline (*b*) and LTP (*c*) for input *S*0. The times are centred on 10 min averages. Analysis of heterosynaptic potentiation (*d*) and total potentiation (*e*) for input S1. (*f–j*) Equivalent experiments when ryanodine was applied during the wTBS.

The effects of ryanodine on STC are illustrated in figure 5. Ryanodine application did not impact baseline synaptic responses at input *S*1 when evaluated at *t* = 35 min, prior to the delivery of sTBS (figure 5*b*). When ryanodine was applied during the sTBS, LTP2 was inhibited at input *S*0 (figure 5*a,c*), consistent with the observations reported in figures 1*g*–*i* and 3*b,d,e*. The levels of LTP, quantified 2 h after the third TBS episode, were 155 ± 4% (*n* = 7) and 137 ± 4% (*n* = 6) in control and ryanodine conditions, respectively (*p* = 0.008; figure 5*a,c*).

Compared with controls, treatment with ryanodine also reduced LTP at input *S*1. At *t* = 175 min, the level of LTP was 136 ± 6% (*n* = 7) and 110 ± 5% (*n* = 6), respectively (*p* = 0.009; figure 5*a,e*). The level of LTP induced at input *S*1 following the sTBS (at input *S*0) in the presence of ryanodine (*t* = 20–70 min) was not significantly different from that induced without prior sTBS application to input *S*0 under control conditions (*p* = 0.447). Also, in ryanodine-treated slices, there was no obvious input *S*1 heterosynaptic potentiation effect associated with the delivery of sTBS at input *S*0. In other words, when ryanodine was applied during the sTBS, this treatment eliminated heterosynaptic effects as compared with controls (*p* = 0.001, *t* = 85 min, figure 5*d*).

In a second set of testing, ryanodine was applied to begin 10 min after the sTBS delivery to input *S*0 and extending past the wTBS delivery to input *S*1 (*t* = 70–120 min; figure 5*f*–*j*). Baseline responses (input *S*0) at *t* = 35 min, pre-ryanodine application, were unaffected (figure 5*g*). For this delayed start of treatment, compared with controls, there was a trend towards lower LTP in ryanodine-treated slices; 162 ± 5% (*n* = 8) and 140 ± 8% (*n* = 9), respectively (*p* = 0.072; figure 5*f,h*).

At the *S*1 pathway, pre-established heterosynaptic potentiation could not be reversed within the first 20 min of ryanodine introduction (figure 5*f,i*). Then, a wTBS was delivered to input *S*1 (figure 5*f*) and led to inhibition of LTP at this input; the final level of LTP was 157 ± 10% (*n* = 8) and 127 ± 6% (*n* = 9, respectively; *p* = 0.035; figure 5*f,j*). This reduced level of LTP at *S*1 input was similar to the LTP observed when wTBS was delivered under control conditions to input *S*1 without any prior sTBS at input *S*0 (*p* = 0.093). In other words, ryanodine eliminated the ‘synaptic capture’ when present during the primed wTBS.

### (f) CPA prevents STC

**Figure 6.**
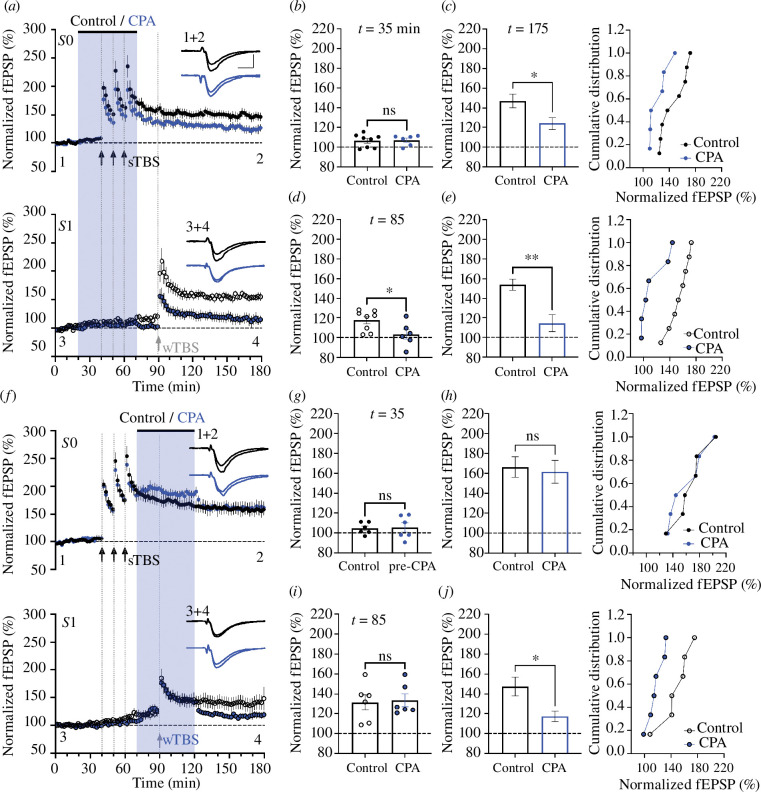
Role of sarcoendoplasmic reticulum calcium ATPase (SERCAs) in heterosynaptic potentiation and STC. (*a*) Time-course plot showing that 30 µM CPA, applied during the sTBS (light blue period), inhibits homosynaptic LTP (at input *S*0) and also heterosynaptic potentiation and STC (input *S*1). Analysis for baseline (*b*) and LTP (*c*) for input *S*0. Analysis of heterosynaptic potentiation (*d*) and total potentiation (*e*) for input *S*1. (*f–j*) Equivalent experiments when CPA was applied during the wTBS.

CPA had essentially the same effects on STC as ryanodine. When CPA (30 µM) was applied during the sTBS, there was no effect on baseline responses (figure 6*d*); however, the level of LTP at *S*0 input was reduced from 147 ± 7% (*n* = 8) to 124 ± 6% (*t* = 175 min; *n* = 6; *p* = 0.034; figure 6*a,c*), similar to the effect illustrated in figure 2*g–i*. CPA applied over this time interval also inhibited LTP induced by wTBS at the *S*1 input, as 90 min following wTBS the levels were 154 ± 6% (*n* = 8) and 114 ± 9% (*n* = 6), in control and CPA conditions, respectively (*p* = 0.001; figure 6*a,e*). Also, in CPA-treated slices, there was no obvious *S*1 input heterosynaptic potentiation effect associated with the delivery of sTBS at input *S*0. Thus, when CPA was present during the sTBS, it eliminated heterosynaptic potentiation (*p* = 0.036, figure 6*d*).

In a second set of experiments, CPA was applied to begin 10 min after the delivery of the sTBS to input *S*0 and to extend 30 min past the delivery of wTBS to input *S*1 (figure 6*f*–*j*). Unlike ryanodine, CPA elicited an acute, but fully reversible potentiation. CPA did not affect the level of LTP2 at input *S*0; quantified 2 h following the last TBS episode, the values were 166 ± 10% (*n* = 6) and 162 ± 11%, in control and CPA conditions, respectively (*n* = 6; figure 6*f,h*, *p* = 0.770). However, CPA inhibited the LTP at input *S*1 induced by the wTBS; 148 ± 9% (*n* = 6) to 117 ± 5% (*n* = 6; *p* = 0.018; figure 6*f,j*). This reduced level of LTP at input *S*1 was indistinguishable from the LTP observed when wTBS was delivered under control conditions to input *S*1 without any prior sTBS at input *S*0 (*p* = 0.395). In summary, CPA inhibited LTP2, heterosynaptic potentiation and STC. The selective effect of CPA on the primed input mirrors the effect observed with ryanodine.

### (g) The relationship between heterosynaptic potentiation and STC

**Figure 7.**
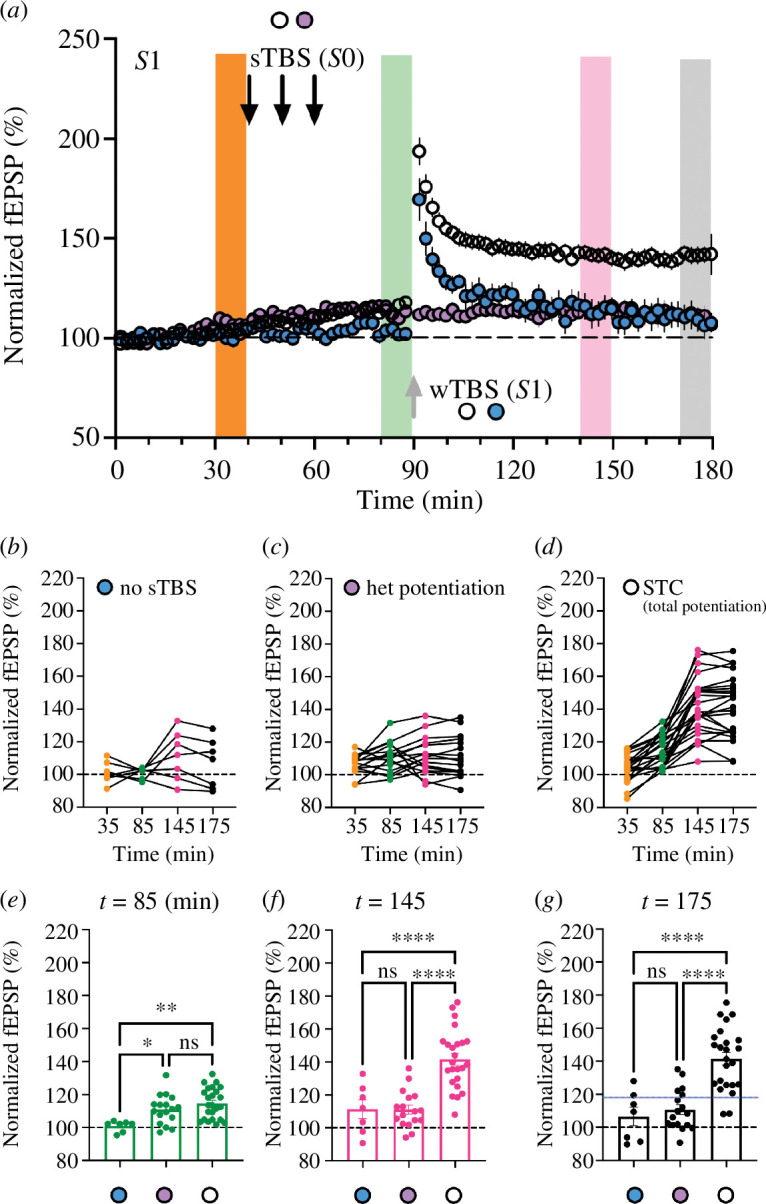
The relationship between heterosynaptic potentiation and STC. (*a*) Superimposed time-course plots at the *S*1 input from all of the experiments involving an unprimed wTBS (blue; *n* = 7), a primed wTBS (open; *n* = 24) and no wTBS at a primed input (i.e. heterosynaptic potentiation; purple; *n* = 19). The coloured doric columns refer to the time range of analyses in (*b*)–(*g*). (*b–d*) Plots of each individual type of experiment at various time points, illustrating the levels of (*b*) unprimed LTP, (*c*) heterosynaptic potentiation and (*d*) total potentiation (which comprised unprimed LTP + heterosynaptic potentiation + STC). The four time points are 10 min averages of normalized fEPSP slopes centred around the times indicated. (*e–g*) Plots of the measurements at specific time points to compared each condition, including (*e*) immediately prior to wTBS at input *S*1, and at two time points following the wTBS (*f,g*). Note that the heterosynaptic potentiation once induced, remains stable throughout (purple in *a*, *e–g*). Note also that the total level of potentiation (white) is much greater than the level of unprimed LTP (blue) + heterosynaptic potentiation (purple), the sum of which is denoted by the horizontal blue–purple dashed line in (*g*).

A complicating factor in our experiments is that the priming induced by the sTBS (input *S*0) resulted in a variable amount of heterosynaptic potentiation (input *S*1). The presence of heterosynaptic potentiation is not unique to our experiments but has been noted in previous STC studies [18], including the original report [19]. An analysis to address the relationship between heterosynaptic potentiation and STC appears in figure 7*a*, which illustrates the time-course in the *S*1 pathway for (i) an unprimed wTBS (i.e. not preceded with a sTBS to input *S*0; *n* = 7, blue circles); (ii) heterosynaptic potentiation (*n* = 19, purple circles); and (iii) the total STC effect (*n* = 24, white circles). The data in figure 7*b–d* display each individual experiment at four-time points: prior to sTBS delivered to input *S*0 (a 10 min average centred at *t* = 35 min; see orange range in figure 7*a* from 30 to 40 min), prior to wTBS to input *S*1 (*t* = 85 min, green corresponding range); and at two periods following the wTBS to input *S*1 (*t* = 145 min, pink; and at 175 min, grey range). The unprimed wTBS (figure 7*b*) was near the threshold for inducing LTP, with 4/7 (57%) slices showing potentiation that declined with time (grand mean of 111 ± 6% at 145 min and 107 ± 6% at 175 min; see figure 7*f,g* for averages and statistical comparisons). In this experiment that received no sTBS at input *S*0 (only baseline stimulation), there was no heterosynaptic potentiation generated at the *S*1 pathway (101 ± 1% at *t* = 85 min; figure 7*b,e*) and hence show that baseline responses were stable.

In contrast, delivery of sTBS (at input *S*0) resulted in a small, variable heterosynaptic potentiation (average of 111 ± 2%, measured at *t* = 85 min; figure 7*c,e*) that persisted for the remainder of the experiment (111 ± 3% at *t* = 145 and 111 ± 3% at 175 min; figure 7*c,f,g*). Delivery of a primed wTBS (i.e. a wTBS delivered to input *S*1 at 30 min following the final sTBS episode at input *S*0) led to potentiation in 19/24 slices (79%) and a greatly enhanced and stable average LTP (of 142 ± 4% and 141 ± 4% at *t* = 145 and 175 min, respectively; figure 7*d,f,g*). Thus, the LTP levels in these STC experiments were considerably greater than what can be accounted for by the heterosynaptic potentiation alone. The group comparison of the three experimental conditions at each of the three final time windows (figure 7*e–g*) allowed statistical comparisons to be performed. As illustrated by a dashed line in figure 7*g*, the sum of the unprimed LTP at input *S*1 (blue) and the heterosynaptic potentiation (purple) reflects roughly half of the total potentiation generated at *t* = 175 min. In summary, the total potentiation at input *S*1 following priming (at input *S*0) comprises LTP1 (i.e. induced in the absence of priming) plus heterosynaptic potentiation plus heterosynaptic metaplasticity.

### (h) Paired-pulse facilitation

Throughout all experiments, the paired-pulse ratio (PPR) was calculated. In response to the delivery of sTBS (e.g. vehicle experiment, electronic supplementary material, figure S1*a*, upper), there was an initial decrease in the corresponding PPR that was associated with STP (electronic supplementary material, figure S1*a*, lower). This change in PPR recovered as STP declined, but remained below pre-TBS values in the grand pools of all wTBS, cTBS and sTBS experiments (electronic supplementary material, figure S1*b–d*; quantified at *t* = 95 min for wTBS and at *t* = 175 min for cTBS and sTBS; *n* = 10, 11, 18 and *p* = 0.015, 0.012, 0.028, respectively).

Baseline PPR did not change upon the addition of CPA (electronic supplementary material, figure S1*f*; *n* = 6, *p* = 0.470), IEM (electronic supplementary material, figure S1*h*; *n* = 8, *p* = 0.534), ryanodine (electronic supplementary material, figure S1*i*; *n* = 7, *p* = 0.804) or a combination of ryanodine and IEM (electronic supplementary material, figure S1*j*; *n* = 7, *p* = 0.689) as evaluated at *t* = 35 min. A small reduction in PPR occurred after the sTBS, which remained statistically significant in two instances (*t* = 175 min, electronic supplementary material, figure S1e*–j*; IEM, *n* = 8, *p* = 0.044; and ryanodine, *n* = 7, *p* = 0.046).

Following the grouping of all STC experiments (electronic supplementary material, figure S2) for the homosynaptic LTP (at *S*0 input; electronic supplementary material, figure S2*a*) and the corresponding heterosynaptic potentiation (at the *S*1 input; electronic supplementary material, figure S2*b*), a statistically significant decrease in PPR emerged again after delivery of the sTBS (*t* = 175 min; *n* = 30, *p* < 0.001; electronic supplementary material, figure S2*c*). Surprisingly, following the sTBS to input *S*0, there was a small but significant reduction in PPR at the *S*1 pathway (*t* = 85 min, purple range; *n* = 30, *p* = 0.001; electronic supplementary material, figure S2*d*). Notably, however, there was no additional change in the PPR following the primed wTBS (after STP had decayed; *t* = 175 min, *S*1 input; *n* = 30, *p* = 0.099).

## 4. Discussion

The main findings of the present study are that Ca^2+^ release from intracellular stores is required for: (i) LTP; (ii) heterosynaptic potentiation; and (iii) STC. This conclusion is based on the use of two compounds that target Ca^2+^ stores in different ways using concentrations that have been used extensively in previous studies of synaptic plasticity [12,13,29,30,34,35]. Both compounds affected the same three processes, LTP2, heterosynaptic potentiation and STC, without affecting significantly several other synaptic processes, including baseline transmission, paired-pulse facilitation, STP and LTP1. Therefore, off-target effects of ryanodine and CPA appear to be unlikely. Future studies could confirm these results using ryanodine receptor 3 knock-out mice [36].

### (a) Role of Ca^2+^ stores in LTP

Previous studies suggested that Ca^2+^ stores have the capacity to modify LTP at CA1 synapses in the hippocampus [9–15]. However, their potential to influence LTP under certain conditions but not others is difficult to reconcile. Several factors may be responsible for recruiting stores, such as the range of stimulus parameters that have been used. In the present study, the difference between the cTBS and sTBS induction protocols was only in the spacing of the episodes (10 s versus 10 min; 75 stimuli in both cases). Therefore, it can be concluded that spacing is one determinant.

The observation that ryanodine and CPA preferentially targeted a component of LTP that is recruited by sTBS is reminiscent of the effects observed previously with inhibitors of CP-AMPARs [17,18,24,33]. In the present study, the effect of one such inhibitor, IEM-1460, was directly compared with, and used in combination with, ryanodine. The finding that the effects of IEM-1460 and ryanodine are similar, and the combination has no additional effect, strongly suggest that CICR is specifically involved in the CP-AMPAR-dependent component of LTP. This component of LTP is also sensitive to inhibitors of PKA [17] and de novo protein synthesis [17] and is termed LTP2 in this article. In contrast, the LTP that was induced by a cTBS or wTBS was not significantly affected by ryanodine or CPA; these forms of LTP are resistant to inhibitors of PKA and de novo protein synthesis [16,17] and termed LTP1. The effects of ryanodine and CPA on LTP2 suggest an association of CICR with the synaptic protein synthesis signalling or machinery.

In the present experiments, there was a tendency for both ryanodine and CPA to partially inhibit wTBS- and/or cTBS-induced LTP, though this did not reach statistical significance. Previous studies have observed an effect of inhibiting CICR on LTP under conditions that would not be expected to induce LTP2. It seems likely, therefore, that in addition to the spacing of the induction protocol, other factors also contribute. One likely possibility is the level of activation of neuromodulators that regulate PKA activity, which could vary between experimental conditions.

Why would CICR be required for LTP2 but not necessarily be required for LTP1? With respect to LTP1, it seems likely that the Ca^2+^ that permeates NMDARs is sufficient, presumably because its primary target, CaMKII, becomes bound to the GluN2B subunit of NMDARs when activated [37]. With respect to LTP2, there are additional steps needed to trigger de novo protein synthesis, one or more of which could require the spread of Ca^2+^ beyond the NMDAR nanodomain. One possibility is that the Ca^2+^-dependent adenylyl cyclases (i.e. AC1 and/or AC8), that generate cAMP to trigger the activation of PKA for LTP2, need CICR for their activation.

Another question is how CICR is triggered at synapses. The synaptic activation of NMDARs can trigger CICR and this does not require activation of AMPARs [5,38–42], though there may be a contribution from metabotropic glutamate receptors (mGluRs) [38]. It seems likely, therefore, that some ryanodine receptors are located on smooth ER close to NMDARs, so as to be able to initiate the CICR cascade. Indeed, the synaptic activation of NMDARs leads to very rapid changes in ER content of dendritic spines [41] via a mechanism that seems to be specific to activated spines [43]. It should be noted that while Ca^2+^ permeation via CP-AMPARs is not required to trigger CICR at synapses [5], it remains a possibility that CICR is involved in the signalling that is initiated by these receptors.

Unlike LTP1, LTP2 can be associated with a small heterosynaptic potentiation, which also requires the activation of CP-AMPARs [18,24,33]. Heterosynaptic potentiation was observed in many but not all control experiments, but was never observed in the presence of ryanodine or CPA, indicating that heterosynaptic potentiation also involves CICR. When induced, heterosynaptic potentiation remained stable for the duration of the experiments (figure 7*a,c,g*), and thus equates to a form of *heterosynaptic potentiation*. It seems plausible that CICRs may provide the mechanism to propagate a Ca^2+^ signal beyond the activated synapses to trigger the heterosynaptic plasticity locally. Building upon this idea, heterosynaptic potentiation could be explained by (i) the spread of cAMP beyond the activated synapses to engage PKA and drive CP-AMPARs into the perisynaptic membrane and (ii) the spread of Ca^2+^, via CICR, to these non-activated synapses to drive said CP-AMPARs into the synapse, potentially via the activation of CaMKK/CaMKI [44].

### (b) CP-AMPARs and STC

In previous work, evidence was provided that CP-AMPARs function as the synaptic tag and that they trigger the generation of the PRPs [18,24]. Evidence was also provided that activation of CP-AMPARs is likewise required for the generation of heterosynaptic potentiation [17]. It was proposed that during a sTBS, the first TBS episode drives CP-AMPARs into the perisynaptic membrane from where they can be: (i) driven into the synapse by the subsequent episodes of TBS within the sTBS train to generate homosynaptic LTP2; (ii) driven into nearby synapses that did not receive any TBS to trigger heterosynaptic potentiation; and (iii) remain at perisynaptic locations at other synapses to serve as a synaptic tag and enable LTP2 in response to subsequent wTBS.

It should be noted that this suggested mechanism is a modification of that originally proposed by Frey and Morris [19,23]. In the original scheme, the strong stimulus triggered the formation of PRPs that were then captured at tagged synapses at independent inputs. In this revised scheme, activation of CP-AMPARs at the strong input helps initiate the formation of PRPs, but rather than initiate widespread protein synthesis, the CP-AMPARs act locally to trigger protein synthesis and induce LTP2 at the activated input. It is proposed that the transfer of information to heterosynaptic inputs is caused by the diffusion of signalling molecules that lead to the insertion of CP-AMPARs into perisynaptic sites; these CP-AMPARs constitute the synaptic tag, and fulfil the criteria that have been suggested elsewhere [23,28]. It is then proposed that the activation of these tagged synapses by a weak stimulus can drive these CP-AMPARs into the synapse where they initiate local protein synthesis to induce LTP2. Therefore this scheme differs from the classical interpretation of STC in that the synthesis of PRPs is local, not widespread. Therefore there is no capture of diffusing PRPs; rather synapses are ‘captured’ in the sense that the PRPs are synthesized locally in response to a weak stimulus (i.e. ones which in the absence of tagging would not initiate protein synthesis). Although this is a modification of the originally proposed STC mechanism, the manifestation of this form of heterosynaptic metaplasticity is the same as originally described. Accordingly, we have retained the STC terminology, though have used a somewhat different definition of the terms.

In its original description, STC was followed for up to 8 h [19]. In the present study, the STC process was followed for 90 min following the weak stimulus. It is therefore not possible to conclude that the CP-AMPAR mechanism proposed here and elsewhere [18,24] can explain the process at later stages of plasticity. In particular, at later stages of LTP, transcription may be required [45] and the perisynaptic insertion of CP-AMPARs cannot readily explain how mRNA could be directed at the necessary ribosomes. It is also not known whether CP-AMPARs can serve as a tag when the weak stimulus precedes the strong [46].

### (c) Role of Ca^2+^ stores in STC

The present study has presented evidence that CICR is required for STC, both during the sTBS and the following wTBS in an independent pathway. This finding builds upon the observation that prior application of ryanodine can enable STC in response to a weak stimulus that would otherwise just generate STP [30]. Although superficially these results would seem to conflict, they can be reconciled by virtue of the dual action of ryanodine to initially cause release and then deplete Ca^2+^ stores [29]. Supporting this explanation, the priming effects of ryanodine are mimicked by caffeine [30] and the inhibitory effects of ryanodine, described herein, are mirrored by CPA, agents that trigger and deplete CICR, respectively. Therefore, it can be concluded that CICR is a critical component of the STC process.

One difference was noted between the effects of ryanodine and CPA. CPA caused a transient small enhancement of synaptic transmission that was most noticeable when applied after a sTBS (figure 6*f*). One possible explanation is that the sTBS loaded the intracellular stores and CPA by blocking the Ca^2+^ pump, resulted in a net efflux of Ca^2+^ that, in turn, facilitated synaptic transmission. Because ryanodine inhibits CICR directly, it does not have the same effect on cytosolic Ca^2+^.

A question that emerges from this study is what determines the extent to which sTBS, at input *S*0, results in (i) heterosynaptic potentiation and (ii) STC, at input *S*1. This point is addressed in figure 7, where the levels of heterosynaptic potentiation and STC are plotted for each individual preparation. In some cases, the level of heterosynaptic potentiation is very small or undetectable and so the majority or entire enhancement of LTP at input *S*1 can be attributed to STC. In one experiment, the heterosynaptic potentiation is sufficient to explain the difference between a primed and unprimed wTBS. In most cases, however, the total effect can best be explained by a roughly equal combination of the heterosynaptic potentiation and STC (figure 7*g*). Accordingly, the LTP at the primed input is a mixture of three phenonema: (i) LTP1 (i.e. the LTP that would be expected to have occurred in the absence of heterosynaptic priming); (ii) heterosynaptic potentiation (a form of LTP2); and (iii) the additional potentiation induced by heterosynaptic metaplasticity. Phenomenon (iii) requires the synaptic tag and so is referred to as STC in the present article (but in many publications there is no distinction made between (ii) and (iii)).

**Figure 8.**
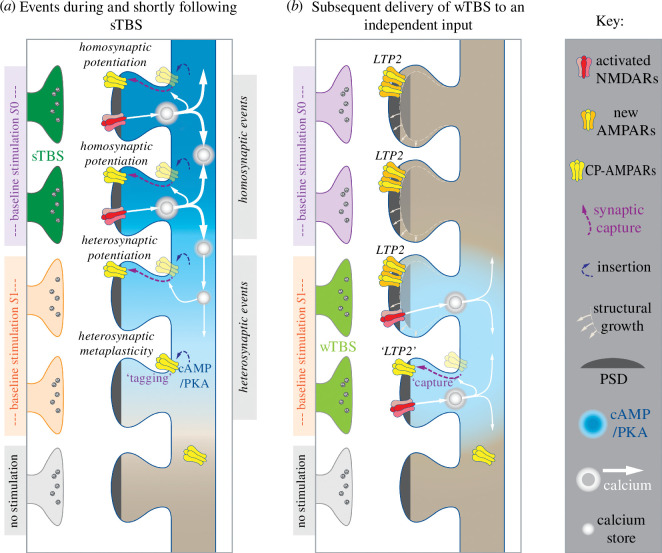
A potential scheme to explain LTP2, heterosynaptic potentiation and STC based on differential spread of cAMP and Ca^2+^. (*a*) Five spines are depicted, of which two are activated by stimulation of input *S*0, two by stimulation of input *S*1 and one unstimulated. These represent a much larger population of intermingled spines on a given dendritic segment. Delivery of a sTBS (‘strong stimulus’) to input *S*0 results in homosynaptic LTP1 (not illustrated) and homosynaptic LTP2. The latter is due to the transient synaptic insertion of calcium-permeable AMPARs (CP-AMPARs) driven by a two-step process involving cAMP/PKA and Ca^2+^. Subsequent baseline stimulation drives Ca^2+^ through these receptors to trigger synapse growth to accommodate a greater number of calcium-impermeable AMPARs (‘new AMPARs’). Critical to heterosynaptic events is the activation of PKA in surrounding synapses, most probably due to the diffusion of cAMP that drives CP-AMPARs into the plasma membrane. In some instances, these are driven into the synapse to generate heterosynaptic potentiation, and in others they remain at perisynaptic sites to tag synapses. Perisynaptic CP-AMPARs are driven into the synapse during the sTBS only when there is a Ca^2+^ signal. For heterosynaptic potentiation, this Ca^2+^ signal is generated by CICR. (*b*) At sites where CP-AMPARs are synaptically inserted, subsequent baseline stimulation drives Ca^2+^ through these receptors to trigger synapse growth to accommodate a greater number of AMPARs. This results in a stable LTP2 at homo- and heterosynaptic sites. At tagged synapses (i.e. where CP-AMPARs are perisynaptically located, i.e. the spine in the fourth row), a subsequent wTBS (‘weak stimulus’) is able to drive them into the synapse to trigger LTP2 in exactly the same manner as above. This process again requires CICR. Therefore, the fundamental difference between homosynaptic and heterosynaptic potentiation on the one hand and STC on the other is the temporal association of the cAMP and CICR-mediated Ca^2+^ signals. (For simplicity AMPAR clusters involved in basal synaptic responses and LTP1 are not illustrated. Only NMDAR clusters that are activated during the TBS are illustrated). Note that CICR could have several roles within synaptic plasticity and metaplasticity, including: (i) to drive the formation of cAMP via activation of Ca^2+^-sensitive adenylyl cyclases; (ii) to drive CP-AMPARs from perisynaptic to the synaptic sites by activation of a CaMK; and (iii) to help trigger de novo protein synthesis.

A hypothetical scheme that addresses the relationship between heterosynaptic potentiation and STC is presented in figure 8. It is proposed that heterosynaptic potentiation is caused by: (i) diffusion of cAMP to activate PKA and drive CP-AMPARs into the perisynaptic plasma membrane of neighbouring synapses; and (ii) a Ca^2+^ signal, initiated by the sTBS, that propagates via CICR to drive these CP-AMPARs into the synapse. Importantly, at some synapses, there is a spread of cAMP beyond the sphere of influence of CICR such that the CP-AMPARs are inserted into the perisynaptic membrane but are not driven into the synapse. Here they tag synapses for a limited period of time before they are removed. Upon a wTBS, the activation of NMDARs drives the perisynaptic CP-AMPARs into the synapse. The variable extent of heterosynaptic potentiation versus STC can be explained by the extent to which CICR matches the region of dendrite that is influenced by the spread of cAMP.

When applied after the sTBS, ryanodine and CPA had little or no effect on homosynaptic LTP (at input *S*0), suggesting that the time window for triggering de novo protein synthesis at input *S*0 had passed. Accordingly, the observation that CICR is also required during the subsequent wTBS to input *S*1 suggests that CICR is required not just for ‘synaptic tagging’ but also for the ‘synaptic capture’ (i.e. the generation of LTP2 at input *S*1). An alternative though less likely explanation is that ryanodine and CPA somehow disrupt the tag, perhaps through the induction of a form of metaplastic depotentiation. Since ‘capture’ cannot happen in the absence of a ‘tag’, the priming of LTP is prevented.

If the former assumption is correct, there is the question as to the role of CICR following the wTBS. There are several possible, non-exclusive, explanations. It is possible that CICR is required for the NMDARs, activated during the wTBS, to initiate the synaptic insertion of CP-AMPARs. Another possibility is that CP-AMPARs require the activation of CICR to trigger protein synthesis. Possibly the Ca^2+^ entry through CP-AMPARs requires magnification to engage the protein synthesis pathway that involves PI3K and ERK [47]. This above model relies on Ca^2+^ gradients and Ca^2+^ nanodomains to separate the activation of AC1/8 and CaMKII and other Ca^2+^-sensitive components in the system. Further studies are required to address this issue more directly.

The model presented in figure 8 is based on a postsynaptic mechanism. However, additional presynaptic changes cannot be excluded, given the need for de novo protein synthesis to strengthen the presynapse and the potential role of CICR in presynaptic mechanisms. In the present experiments, the test stimuli were delivered as paired pluses (50 ms interval) and the PPR was calculated for all experiments. Initially following the TBS there was a decrease in PPR, which is most likely explained mainly by an increase in the probability of release, P(r), that is associated with STP [32]. By the time that LTP had stabilized, a small decrease in PPR persisted. PPR is a complex measure that may also reflect postsynaptic alterations due to, for example, the transient exchange of CP-AMPARs for calcium-impermeable AMPARs and alterations in synaptic inhibition. Therefore it is not possible to ascertain whether the small alterations in PPR are due to changes in P(r) or not. Where there is no change in PPR, it is most likely that P(r) is unaffected. Since there was no persistent change in PPR following the primed wTBS, the most likely explanation is that STC, as studied under the present conditions, is a postsynaptic phenomenon.

### (d) The relationship between homosynaptic and heterosynaptic metaplasticity

In addition to STC, which is a heterosynaptic form of metaplasticity, homosynaptic metaplasticity at these synapses is also well documented [48,49], and also involves CICR [12,13,29]. Early examples of homosynaptic metaplasticity include the priming action of mGluRs to augment subsequent LTP [48,50,51] via generation of de novo protein synthesis [52]. An intriguing concept arises regarding the role of mGluRs in STC and what factors determine whether metaplasticity is homosynaptic or heterosynaptic in nature. In principle, CP-AMPARs could be the sole determinants of the latter. Further experiments will be required to address the relationship between CP-AMPARs and mGluRs in STC.

### (e) Concluding remarks

The present study has provided evidence that CICR is required for the same synaptic mechanisms that also involve CP-AMPARs. This equates to the same synaptic processes that also involve PKA and de novo protein synthesis. These processes involve both homosynaptic (i.e. LTP2) and heterosynaptic (i.e. heterosynaptic potentiation and STC) plasticity. It is suggested that CICR is a critical component for these heterosynaptic mechanisms to occur, associative processes that may underlie long-term memory formation.

## Data Availability

This article has no additional data. Representative samples of the raw electrophysiological field responses are included herein, and full sweeps from all time-course experiments are available upon request. Electronic supplementary material is available online at [53].
